# Revealing the Mutation Patterns of Drug-Resistant Reverse Transcriptase Variants of Human Immunodeficiency Virus through Proteochemometric Modeling

**DOI:** 10.3390/biom11091302

**Published:** 2021-09-02

**Authors:** Jingxuan Qiu, Xinxin Tian, Jiangru Liu, Yulong Qin, Junjie Zhu, Dongpo Xu, Tianyi Qiu

**Affiliations:** 1 School of Medical Instrument and Food Engineering, University of Shanghai for Science and Technology, Shanghai 200093, China; jxqiu@usst.edu.cn (J.Q.); tianxx0511@163.com (X.T.); Liurlilkey@163.com (J.L.); qyl1218@139.com (Y.Q.); hjkkhhh@163.com (J.Z.); xudongpo@usst.edu.cn (D.X.); 2 Shanghai Public Health Clinical Center, Fudan University, Shanghai 200032, China

**Keywords:** HIV, reverse transcriptase, drug resistance, computational model

## Abstract

Drug-resistant cases of human immunodeficiency virus (HIV) nucleoside reverse transcriptase inhibitors (NRTI) are constantly accumulating due to the frequent mutations of the reverse transcriptase (RT). Predicting the potential drug resistance of HIV-1 NRTIs could provide instructions for the proper clinical use of available drugs. In this study, a novel proteochemometric (PCM) model was constructed to predict the drug resistance between six NRTIs against different variants of RT. Forty-seven dominant mutation sites were screened using the whole protein of HIV-1 RT. Thereafter, the physicochemical properties of the dominant mutation sites can be derived to generate the protein descriptors of RT. Furthermore, by combining the molecular descriptors of NRTIs, PCM modeling can be constructed to predict the inhibition ability between RT variants and NRTIs. The results indicated that our PCM model could achieve a mean AUC value of 0.946 and a mean accuracy of 0.873 on the external validation set. Finally, based on PCM modeling, the importance of features was calculated to reveal the dominant amino acid distribution and mutation patterns on RT, to reflect the characteristics of drug-resistant sequences.

## 1. Introduction

According to the World Health Organization, there are 38 million people worldwide living with human immunodeficiency virus (HIV) in 2019 [1]. Moreover, approximately 33 million deaths have been reported due to HIV [1], which remains a major global public health issue. In the clinical treatment of HIV-1 infection, most drugs target enzymes, including protease (PR), reverse transcriptase (RT), and integrase (IN) [2]. HIV reverse transcriptase (RT) is a common target in highly active antiretroviral therapy, and RT inhibitors can target the early stages of virus-host interactions [3]. The lack of proofreading capability of HIV RT combined with a high replication rate leads to a wide range of genetic variability [4], resulting in drug resistance. The rapid emergence of drug-resistant virus variants is an obstacle to the success of anti-HIV agents. Drugs approved by the U.S. Food and Drug Administration (FDA) for RT variants include nucleoside reverse transcriptase inhibitors (NRTI) and non-nucleoside reverse transcriptase inhibitors (NNRTI) [5]. HIV-1 RT is composed of a heterodimer of the p66 subunit and p51 subunit, DNA-binding pocket, and active sites located on the p66 subunit [6]. Thus, the p66 sequence variation should be considered in RT drug design.

Several studies have demonstrated that the existing drug resistance could affect the treatment regimen and genotyping and drug-resistance tests are recommended before starting clinical therapy [7,8]. To deal with drug resistance, several approaches, including resistance prediction and a combination of drugs, have been tested. Several in silico HIV resistance prediction models and computer-aided drug design (CADD) have been proposed for the development of drug design. Beerenwinkel et al. proposed an information profile to interpret the sequence variation of PR and RT sequences, and decision tree classifiers were constructed to predict resistance or susceptibility to drugs [9]. Thereafter, a regression model was generated to predict phenotypic drug resistance based on 650 genotype-phenotype pairs [10]. To optimize the use of existing drugs and understand the genetic basis of drug resistance, Rhee used five statistical learning methods to determine the correlation between mutations in the protein (PR/RT) sequence and the susceptibility of 16 antiretroviral drugs. The accuracy tested by 5-fold cross-validation reached 80.1% [11].

Based on the amino acid sequence of the target protein, several descriptors have also been designed and used in machine learning model construction. Tarasova et al. [12] used short fragments of both amino acid sequences and nucleotide sequences as descriptors. The performance of the two descriptors was compared using the random forest algorithm for model construction. By constructing the drug-specific resistance prediction model, it was demonstrated that the model performance was more sensitive to drug type than the descriptors. Agata Paneth et al. [13] constructed a quantitative structure-activity relationship (QSAR) model based on the docking results of 47 inhibitors to 107 allosteric centers. Shiri et al. [14] calculated the 2D and 3D molecular descriptors and fingerprints for NNRTIs and then used a genetic algorithm to select variables. The support vector machine model was constructed based on the designed molecular descriptors with the EC_50_ values to classify the compounds into active and inactive ones. Furthermore, weighted categorical kernel functions were introduced to evaluate the contribution of different positions on the resistance prediction [15]. Recently, Brand expanded the application of the prediction model and proposed a multi-label classification model to predict the cross-resistance between RT sequences and five nucleoside analogs [16].

These machine learning approaches could provide a rapid and accurate prediction of drug-target relationships and are helpful in virtual screening in drug design. In recent studies, more and more researchers have proposed that the feature importance should be determined to increase the explanation of machine learning approaches [17]. In this study, an in silico random forest drug-resistance prediction model was proposed to classify the binding potential between nucleoside reverse transcriptase inhibitors (NRTI) and reverse transcriptase (RT), based on the protein descriptors for RT and the molecular fingerprints for NRTI. Further, 40 key features contributing to classification in the prediction model were screened. The mutation patterns and distributions on the selected 10 sites were proposed to illustrate the possible mutations that lead to drug resistance. The proposal of this model could be helpful for drug usage in HIV treatment.

## 2. Materials and Methods

### 2.1. Datasets

The genotype and phenotype data of the reverse transcriptase in this study were derived from the HIV Drug Resistance Database [18]. A total of 1683 non-redundant mutated sequences of reverse transcriptase were collected. The in vitro susceptibility tests were performed using the PhenoSense assay [19], which included 9538 fold resistance values, calculated by dividing the IC_50_ value of the drug for mutated RT by the IC_50_ value of the drug for the wild-type RT [12]. The tested drugs included lamivudine (3TC), abacavir (ABC), zidovudine (AZT), stavudine (D4T), didanosine (DDI), and tenofovir (TDF). For each drug, the resistant variants and susceptible variants were classified based on the cutoff of fold resistance [12]. The detailed cutoffs combined with the numbers of each class are listed in Supplementary Table S1. The full data set of 9538 fold resistance values, including 5317 susceptible variants and 4221 resistant variants, was randomly split into 7625 training datasets (80%) and 1913 independent testing datasets (20%) without changing the proportion of the two classes (Table S2). The random splitting of the training and testing datasets was evaluated 10 times.

### 2.2. Protein Structure Modeling

To illustrate the spatial features and compare the structural deviations between the consensus RT and RT mutants, the three-dimensional structure of RT proteins was constructed using SWISS-MODEL [20]. The consensus RT sequence was obtained from the HIV Drug Resistance Database [18]. For homology modeling, the template structure of RT was derived from the Protein Data Bank [21] by searching for the ID of 4ZHR (Chain A). The sequence identity between consensus RT sequence and template was over 98.6%, with a sequence coverage of 100%. Thereafter, the spatial structure of the RTs can be constructed, and the pdb file, which contains the three-dimensional coordinates of each atom, can be obtained.

### 2.3. Mutation Sites Selection

To describe the mutated reverse transcriptase, important sites with frequent mutations were screened. For 1683 reverse transcriptase sequences, among the total length of 562 amino acids, there were 372 sites on which mutations occurred, including insertion, deletion, and mutation. The mutation sites were defined as sites with a mutation frequency of more than 10% among all sequences. Finally, 47 frequent mutation sites were selected for protein description (Table S3).

### 2.4. Ligand Binding Site Prediction

To detect the relationship between selected mutation sites and potential ligand-binding sites for RT protein, the constructed RT structure was uploaded to POCASA [22] to predict the potential ligand-binding sites under the default parameters. The atoms involved in the ligand-binding sites were listed and divided into different regions based on their spatial locations.

After determining all potential ligand-binding sites on the RT structure, the spatial relationship between mutation sites and potential ligand-binding sites was measured using Euclidean Distance *ED(s_j_)*, as shown in Equation (1):

(1) $$ED\left(s_{j}\right)=\min\left\{\sqrt{{\left(X\left(s_{jk}\right)-X\left(l_{m}\right)\right)}^{2}+{\left(Y\left(s_{jk}\right)-Y\left(l_{m}\right)\right)}^{2}+{\left(Z\left(s_{jk}\right)-Z\left(l_{m}\right)\right)}^{2}}\;\right\}$$

where *ED(s_j_)* refers to the minimum distance between site *j* (*s_j_*) and all potential ligand-binding sites. *s_jk_* refers to atom *k* in residue *s_j_*, and X(*s_jk_*), Y(*s_jk_*), and Z(*s_jk_*) refer to the spatial coordination of atom *k* in residue *s_j_* X(*l_m_*), Y(*l_m_*), and Z(*l_m_*) refer to the spatial coordination of atom *m* in the predicted ligand-binding atom. Then, the minimum distance between any selected mutation sites and ligand-binding sites can be calculated.

### 2.5. Descriptor Generation

The drug resistance descriptor was composed of two parts: a protein descriptor and a drug descriptor. A protein descriptor was designed to describe the changes in the properties of amino acid mutations. All mutations were compared to the consensus sequence of the reverse transcriptase from the HIV Drug Resistance Database [18]. The Z-scales were designed to describe the protein features (Table S4), which were the result of principal component analysis (PCA) from the initial 26 physicochemical descriptors [23]. The 26 variables include retention values in chromatography, nuclear magnetic resonance shift, van der Waals volume, nonpolar surface area, hydrogen bond donor, side-chain charge, and so on. After PCA transformation, the final Z1–Z5 scores were calculated, and the detailed interpretation is as follows:

(1). Z1: lipophilicity scale. Negative Z1 refers to lipophilic residues, and positive Z1 correlates to hydrophilic ones.
(2). Z2: steric bulk, molecular weight and van der Waals volume.
(3). Z3: description of polarity.
(4). Z4 and Z5: combined properties, including electronegativity, electrophilicity, and hardness.

It should be noted that the Z scale could provide quantitative scales and translate each residue into descriptors, which cover multiple physicochemical properties.

For the mutations that occurred at 47 sites, protein descriptors were calculated for different mutations. (1) Point mutation: The descriptor score was calculated as the absolute difference between the Z score for mutated amino acids and the Z score for previous amino acids. (2) Deletion: The descriptor score was calculated as the maximum of the absolute difference between the Z score of any amino acid and the Z score of the previous residue. (3) Mixture: The scores were calculated as the absolute difference between the average Z values for the mixture amino acids and the Z value for the previous residue. (4) Insertion: The absolute difference between the Z sum of inserted amino acids and the Z value of the previous residue. The 235-bit protein descriptors were generated from the calculated Z1–Z5 scores for the 47 sites. The 200-bit drug descriptors were constructed using RDKit (release Version 2017). A detailed description of each bit of drug descriptor is listed in Table S5. A total of 435-bit descriptor was generated to build the prediction model.

### 2.6. Model Construction

**Figure 1:**
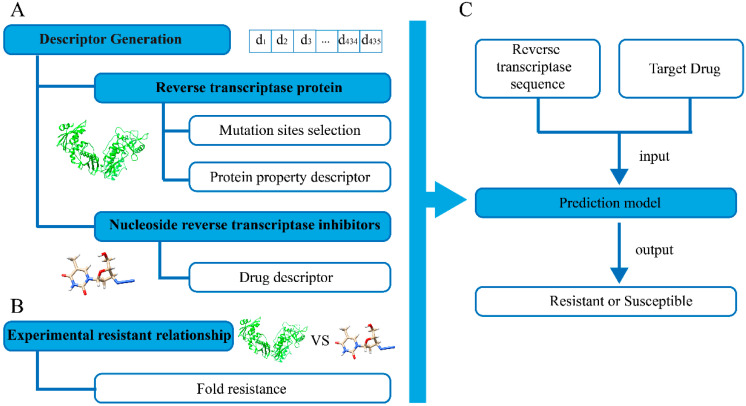
Workflow of drug resistance prediction model for HIV-1 reverse transcriptase. (**A**) The 435-bit descriptors describing the physic-chemical and structural properties of the transcriptase proteins and NRTIs. (**B**) The experimental assay was collected to reflect the resistant or susceptible relationship between transcriptase proteins and NRTIs. (**C**) Machine learning approaches were introduced based on the descriptors and the experimental relationship to generate the PCM model.

To build a computational model to predict drug resistance, different machine learning approaches, including random forest, logistic regression, decision tree, naïve Bayes, and supporting vector machine, have been tested. Based on the descriptors and fold resistance values in the training dataset containing 7625 drug-protein pairs, 10-fold cross-validation was performed to select the machine learning algorithm. The hyperparameters for each tested model are listed in Table S6. By inputting the drug descriptors and reverse transcriptase protein descriptors, the constructed model could predict whether the HIV-1 RT variants were resistant to the drug. The entire workflow to construct the prediction model is shown in Figure 1.

### 2.7. Model Evaluation

The internal and external validation were tested to evaluate the overall performance of the model from different aspects, including AUC value, accuracy, precision, recall, and F-score. The parameter definitions are listed in the following equations:

(2) $$\mathrm{Accuracy}=\frac{TP+TN}{TP+FP+TN+FN}$$

(3) $$\mathrm{Precision}=\frac{TP}{TP+FP}$$

(4) $$\mathrm{Recall}=\frac{TP}{TP+FN}$$

(5) $$F-\mathrm{score}=\frac{2}{1/\mathrm{precision}+1/\mathrm{recall}}$$

the positive and negative samples refer to the drug-susceptible and drug-resistant samples, respectively. *TP* represents the number of true positive samples, *TN* refers to true negative samples, *FN* refers to false negatives, and *FP* refers to false positives.

### 2.8. Calculating Feature Importance

For any feature in the above designed 435-bit descriptors, the feature importance was calculated using the scikit-learn 0.22.1 package in Python 3.8.2 (detailed version can be found in Table S7) and ranked in descending order. The feature importance was calculated by the function “feature_importances” in the scikit-learn package, with Gini importance as the returned value, evaluating data impurity in each node in the forest. The higher the value, the more important the feature is. For any ith feature in the ranking list, the accumulated feature importance (*AF*) and the growth rate of importance (*GR*) can be calculated using Equations (6) and (7):

(6) $$AF\;\left(\;i\;\right)=\overset{i}{\underset{1}{\sum }}f\left(i\right)$$

(7) $$GR\;\left(i\right)=\frac{AF\left(i\right)-AF\left(i-1\right)}{AF\left(i-1\right)}$$

where *f*(*i*) refers to the importance of feature *i, f*(*i*) ranges from 0 to 1, and *i* ranges from 1 to 435. The accumulated *AF* for all 435-bit descriptors was 1. According to the ranking list of feature importance, the mutation sites involved in the top-ranking features with *AF* over 50% were selected as the dominant sites.

### 2.9. Detecting Mutation Patterns in Experimental Pairs

To detect the important characteristics of RTs, the mutation patterns of 5317 drug-susceptible proteins and 4221 drug-resistant proteins were derived and compared using the following steps:

(1) Calculate the residue distribution on the individual target sites. For each target site, the residue frequencies in both experimentally determined drug-susceptible proteins and drug-resistant proteins were calculated.

For any amino acid *i* (*a_i_*) at site *j* (*s_j_*), the absolute difference in the amino acid frequency *DF(a_i_,s_j_)* was defined as Equation (4):

(8) $$DF\left(a_{i},s_{j}\right)=\left|FS\left(a_{i},s_{j}\right)-FR\left(a_{i},s_{j}\right)\right|$$

where *i* represents one of the 20 amino acid types, and *j* represents one of the 47 mutation sites. *DF(a_i_,s_j_)* refers to the absolute difference of the amino acid frequency on the drug-susceptible protein *FS(a_i_,s_j_)* and the frequency of drug-resistant proteins *FR(a_i_,s_j_)*. Then, we calculated the standard deviation of amino acid frequency changes at each site *j*, *SD(s_j_)*, based on the value of *DF(a_i_,s_j_)*.

(2) Deriving the mutation patterns of the target sites. The amino acids on the dominant sites (Section 2.8) were joined as peptide fragments. Then, the distribution of each joint fragment was counted to form the mutation pattern.

### 2.10. Evaluation of Mutation Patterns

Furthermore, we evaluated the mutation patterns detected in our model through molecular docking. In this study, the relationship between all 1683 proteins and six drugs was predicted using our PCM modeling. For all 10,098 pairs (1683 × 6), the relationship for 9538 pairs was determined by previous experiments in the HIV Drug Resistance Database [18], while the remaining 560 pairs lacked experimental evidence.

For the above 560 pairs, new joint peptide fragments (Section 2.9) were detected in the predicted drug-resistant mutants and selected as the potential mutation pattern. Joint peptide fragments that already occurred in the above 9538 experimentally validated pairs were excluded.

To validate the newly detected mutation pattern, we mapped the above peptide fragment on the consensus RT sequence to generate new RT mutants with the new mutation pattern. Thereafter, the three-dimensional structures of each protein mutant were built using SWISS-MODEL [20] (Section 2.2). The drug-binding probability and modes between the new RT mutants and six drugs were predicted by SwissDock [24]. In comparison, the best FullFitness score between each drug and protein was calculated to evaluate the binding ability.

## 3. Results

### 3.1. Spatial Location of Screened Mutation Sites

**Figure 2:**
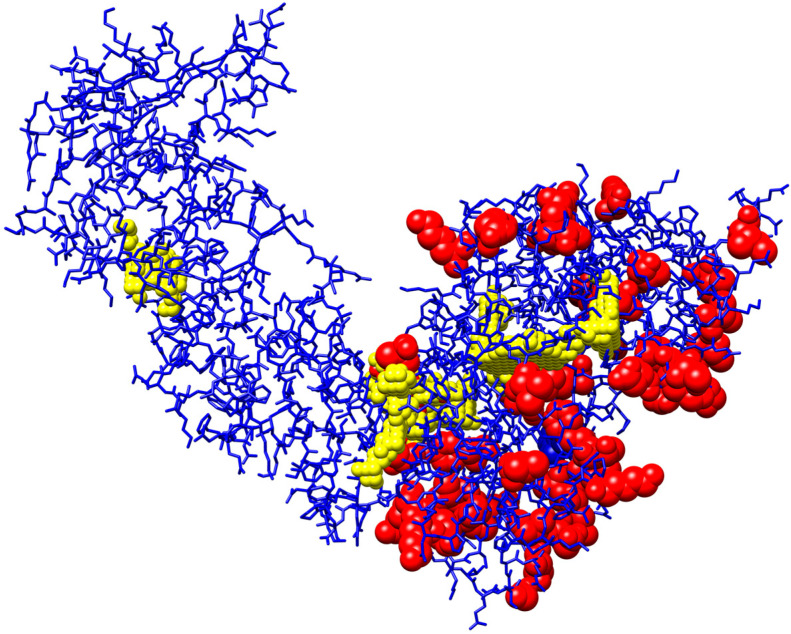
Spatial illustration of key mutation sites. The 3D structure of reverse transcriptase is labeled in blue. Ligand-binding sites predicted by POCASA are labeled as yellow balls, and the 47 key mutation sites in this model are colored as red balls.

For fingerprint generation, 47 amino acid positions with high mutation frequencies were initially screened as the key mutation sites of RT (Table S3). Thereafter, five potential ligand-binding pockets were predicted by POCASA [22], and the spatial structure was presented by Chimera [25] in Figure 2. The relative spatial location between mutation sites and predicted ligand binding sites was measured using Euclidean distance *ED(s_j_)* (Section 2). The detailed distances are listed in Table S8. In general, the nearest distance between mutation sites and atoms in ligand binding sites was 12.47 ± 5.59 Å (mean ± standard derivation). It was found that 61.702% (29/47) of the dominant mutation sites were located within 15 Å around the ligand-binding sites, and the nearest atom distance was only 2.102 Å. Mutations at all these sites could impact the micro-environment, including electronic properties, steric effects, and hydrogen bond donors [5], and affect the performance in the in silico prediction [26].

### 3.2. Model Performance on Drug Susceptibility Prediction

With the designed protein and drug descriptors, five machine learning methods, including random forest (RF), logistic regression (LR), decision tree (DT), naïve Bayes (NB), and support vector machine (SVM), were introduced to construct different PCM models. Through 10-fold cross-validation on 7625 training data, the results of internal validations can be found in Table 1. The results showed that all evaluated approaches could provide satisfactory performance with a mean AUC value over 0.791 and an average accuracy over 0.712 in the 10-fold cross-validation, which indicated that the designed descriptors could provide an accurate description of the physicochemical features of both RT variants and NRTIs. Random Forest achieved the best prediction performance with an average AUC value over 0.921 and an average accuracy over 0.827. Therefore, a random forest classifier was selected to construct the PCM model for resistance prediction.

To compare the performance of different machine learning models, the Mann-Whitney test was used to compare the predicted probability values of resistant drug-protein pairs and those of susceptible drug-protein pairs. The *p*-values of each fold in the validation are listed in Table S9. It was found the *p*-value for all machine learning approaches illustrated statistical significance between the prediction scores for susceptible and resistant pairs.

**Figure 3:**
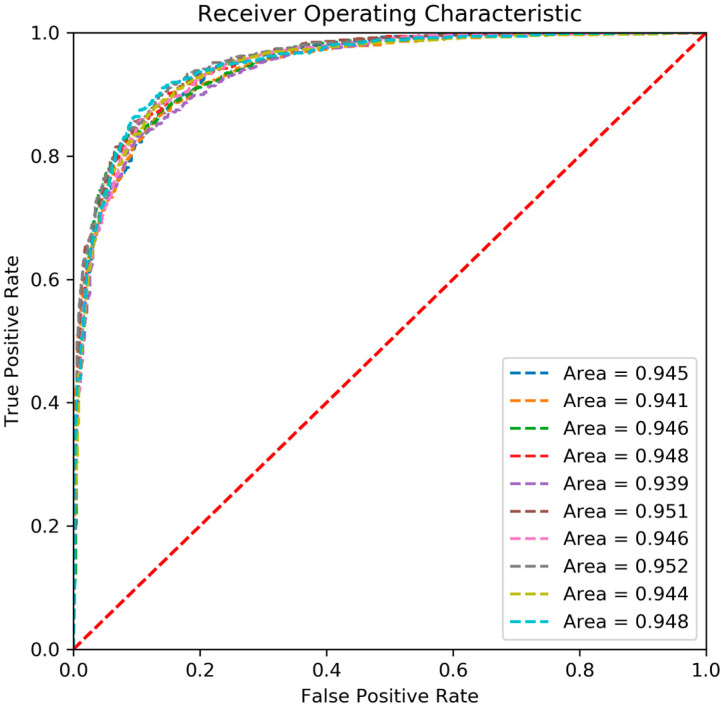
Receiver Operating Characteristic curve of prediction results on the 10 independent validations by Random Forest model.

To illustrate the stability of the model performance, the random forest classifier was tested on an independent testing dataset 10 times (Section 2.1). It was found that the model achieved a stable and high performance with an AUC value of 0.946 ± 0.004 and an accuracy of 0.873 ± 0.007 (Table S10). The ROC curves for the 10 tests are shown in Figure 3, indicating the stable and good performance of our PCM model for predicting drug resistance.

### 3.3. Detecting Important Features for HIV-1 Drug Resistance

**Figure 4:**
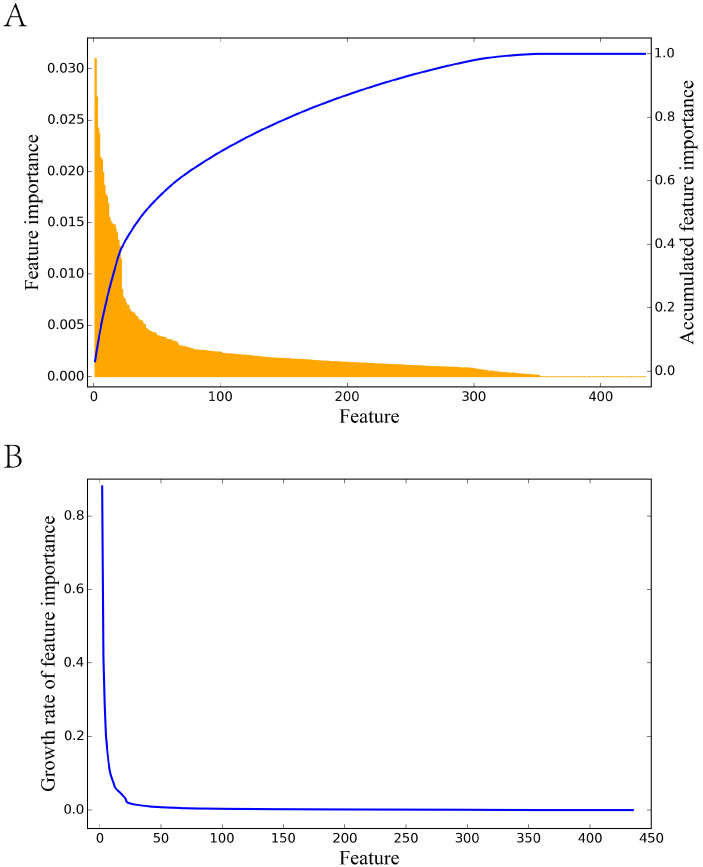
Illustration of feature importance, accumulated importance, and the growth rate of feature importance. (**A**) The orange bar refers to the feature importance of each 425 feature in descriptors. The blue line refers to the accumulated feature importance. (**B**) The growth rate of feature importance.

For computer-aided drug design, the detection of important features of drug resistance is essential. In this study, the features that significantly contributed to drug resistance predictions were screened to reveal the drug resistance patterns. We introduce three parameters: the importance of each feature *i*, *f(i)*, the accumulated importance of features (AF), and the growth rate of feature importance (GR) as indicators of feature contributions (Section 2). As illustrated in Figure 4, the importance of each feature *f(i)* is not even, varying from approximately 0 to 0.031. When ranked in descending order according to feature importance, there are 40 features with GR over 0.01, demonstrating the fast accumulation of contributions for these features in the model. In general, 40 out of 435 features (9.20%) contributed more than 50% of the AF, and these features were selected as the most essential features for HIV-1 drug resistance (Table S11).

Among the 40 important features, 87.5% (35/40) were protein descriptors for different dominant mutation sites; therefore, the mutations in HIV-1 RT sequences have a significant effect on the resistance of NRTIs. For example, the top three features were related to the Z2 score on site 210, Z2 score on site 215, and Z3 score on site 215, respectively. The scale of Z2 is the sum of steric bulk, which reflects the value related to molecular weight, van der Waals volume, and total surface area [23]. The scale of Z3 mainly describes the polarity of each amino acid [23]. Therefore, the structural features of steric bulk and physicochemical properties of amino acids are the most important elements for HIV-1 drug resistance.

**Figure 5:**
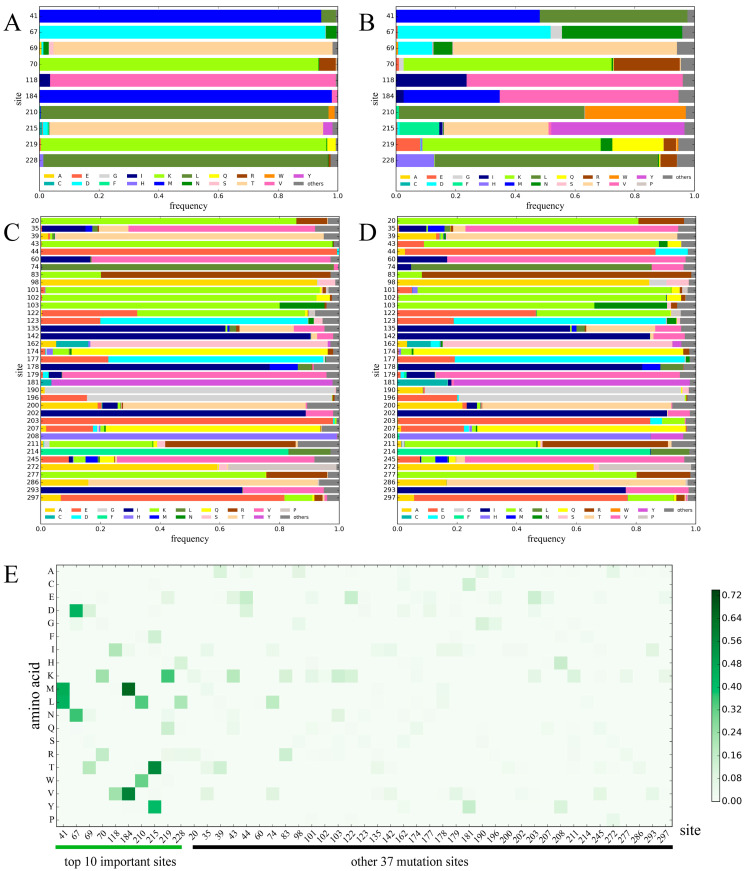
Amino acid distribution and frequency changes of 3TC-susceptible and 3TC-resistant proteins on 47 sites. Sub-graphs (**A**,**B**) show the amino acid frequencies on 10 dominant sites for 3TC-susceptible proteins and 3TC-resistant proteins, respectively. Sub-graphs (**C**,**D**) show the amino acid frequencies on 37 mutation sites for 3TC-susceptible proteins and 3TC-resistant proteins, respectively. Sub-graph (**E**) illustrates the absolute difference of residue frequency on each site between 3TC-susceptible and 3TC-resistant proteins. The X axis refers to each mutation site. The Y axis refers to 20 amino acids. Each pixel refers to the value of *DF(a_i_,s_j_)* for each amino acid *a_i_* on 47 sites *s_j_*. The color of each pixel correlates to the value of *DF(a_i_,s_j_)*.

Further analysis showed that 10 mutation sites were involved in the above 35 protein descriptors, including 41, 67, 69, 70, 118, 184, 210, 215, 219, and 228, which remained to reveal the mutation patterns of drug resistance. The residue frequency on the 10 dominant sites from drug-susceptible and drug-resistant proteins was counted (Figure 5 and Figures S1–S5). Based on the result of drug 3TC (Figure 5A,B), there were 446 drug-susceptible proteins in our dataset, and all contained at least one dominant residue with a frequency of over 90% for each of the above 10 dominant mutation sites. For example, Met (M) on site 41 (frequency 94.395%), Asp (D) on site 67(frequency 95.740%), Thr (T) on site 69 (frequency 93.498%), Lys (K) on site 70 (frequency 93.498%), Val (V) on site 118 (frequency 95.740%), Met (M) on site 184 (frequency 97.982%), Leu (L) on site 210 (frequency 96.413%), Thr (T) on site 215 (frequency 91.480%), Lys (K) on site 219 (frequency 95.740%), and Leu (L) on site 228(frequency 95.292%). However, for 1192 3TC-resistant proteins in our dataset, the frequency of dominant sites decreased sharply, and the frequency of other residues increased synchronously. For some sites, the population of dominant residues in 3TC-resistant proteins remained dominant with a decreased population. For example, at site 219, the population of the dominant residue Lys (K) decreased from 95.740% in 3TC-susceptible proteins to 59.732% in 3TC-resistant proteins, while that of Gln (Q) increased from 2.915% to 17.114%. For the other sites, the dominant residue was shifted. Typical examples are sites 41 and 215. For site 41, the population of Leu (L) was only 4.933% in 3TC-susceptible proteins, which rapidly increased to 49.497% in 3TC-resistant proteins, whereas the previous dominant residue Met (M) in 3TC-susceptible proteins decreased to 48.071% in resistant proteins. A similar situation was observed at site 215, in which the dominant residue Thr (T) in the 3TC-susceptible proteins decreased from 91.480% to 34.899%, and the second Tyr (Y) increased from 3.139% to 44.631% in resistant proteins. The above results showed that the dominant amino acids at key mutation sites are essential for drug resistance. Drug-susceptible proteins tend to contain conservative residues, whereas drug-resistance proteins contain diverse residue compositions.

To illustrate whether the observed pattern on the 10 dominant sites also occurred on the other 37 mutation sites. The residue frequency at 37 mutation sites was also calculated for both 3TC-susceptible proteins and 3TC-resistant proteins (Figure 5C,D). The frequency change at 37 sites is relatively small compared with the 10 dominant positions. To quantitatively test the differences between the top 10 dominant positions and the other 37 mutation sites, the measurements of *DF(a_i_,s_j_)* and *SD(s_j_)* were calculated (Section 2). As shown in Figure 5E and Table S12, both the *DF(a_i_,s_j_)* and *SD(s_j_)* of 10 dominant sites are larger than those of 37 mutation sites with significant *p*-values (Table S13), which refers to larger changes in amino acid frequency at 10 dominant sites.

### 3.4. Mutation Patterns of Joint Fragment on Target Sites

**Figure 6:**
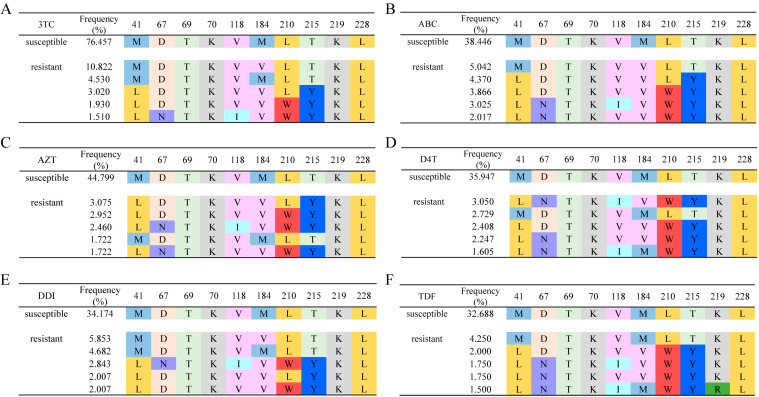
Peptide distribution for drug-susceptible and drug-resistant proteins. Subgraph (**A**–**F**) refers to peptide distribution for 3TC, ABC, AZT, D4T, DDI, and TDF related drugs. On each subgraph, the first line refers to the most frequent peptide on drug-susceptible proteins. The following line refers to the frequently occurred peptide on drug-resistant proteins.

Moreover, by aligning the residues on the above 10 sites, the joined fragments on the dominant sites for different drugs were evaluated, as shown in Figure 6. In drug-susceptible proteins, the joined fragments contained dominant patterns. For example, 76.457% of the 3TC-susceptible proteins had the joined set of MDTKVMLTKL, which was only observed in 4.530% of the 3TC-resistant proteins. For 3TC-resistant proteins, the pattern of the joined fragment was not significantly observed, among which the top 1 joined set MDTKVVLTKL only covers 10.822% of the population. Similar results could also be found in drugs ABC, AZT, D4T, DDI, and TDF, which contain dominant joined residues set with a frequency over 32%, while this frequency was less than 5% for drug-resistant proteins. The results showed the dominant pattern of joined residue fragments in drug-susceptible proteins. Furthermore, mutated proteins contain varied mutations, which might be the reason for drug resistance.

The mutations on the above dominant sites also introduce huge property changes, resulting in a decrease in binding probability. Typical examples such as the mutation from Asp (D) to Asn (N) frequently occurred at site 67, which involved property changes from acidic residues to neutral residues. Mutations from Leu (L) to Trp (W) at site 210 and Thr (T) to Tyr (Y) at site 215 will lead to the introduction of a benzene ring and may result in the change of steric bulk to reduce the binding affinity of the target drugs.

Furthermore, we evaluated the mutation patterns of the predicted resistant proteins in our model. For all 10,098 pairs between 1683 proteins and six drugs, there were a total of 560 drug-protein pairs without experimental results for drug resistance testing. For each of the above pairs, the mutation pattern of the joint fragment on the dominant mutation sites was detected. For each drug, the prevalent mutation patterns detected in at least three proteins are listed in Table S14. In general, 11 protein patterns were detected, and three patterns, including *NTKIVWYNL* for ABC-resistant proteins, *LNTKIMWYKL* for TDF-resistant proteins, and *LNDKIVWYKL* for TDF-resistant proteins were newly detected in the predicted drug-resistant proteins without previous experimental evaluations.

**Figure 7:**
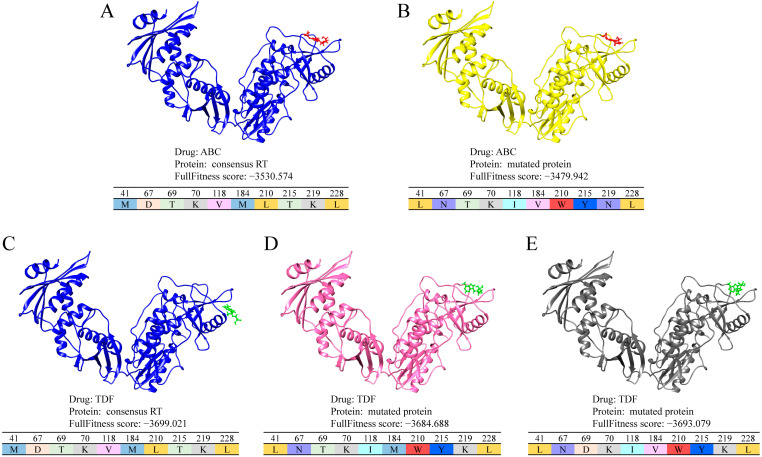
The docking result of consensus RT and mutated protein with the newly detected pattern. (**A**) The docking structure of ABC and consensus RT. (**B**). The docking structure of ABC and mutated protein with new sequence pattern of LNTKIVWYNL on dominant sites. (**C**). The docking structure of TDF and consensus RT. (**D**). The docking structure of TDF and mutated protein, with the new mutation pattern of LNTKIMWYKL. (**E**). The docking structure of TDF and mutated protein with the new mutation pattern of LNDKIVWYKL.

To evaluate the reliability of the above three mutation patterns, the protein variants were mutated based on the consensus RT sequence, and the corresponding protein structures were constructed by SWISS-MODEL [20]. Furthermore, a molecular docking approach was introduced to compare the binding probability of the same drug between the protein variants with mutation patterns and the consensus RT proteins using SwissDock [24]. A lower FullFitness score indicates more stable binding between proteins and drugs. As shown in Table S14, in addition to the score between protein variants 3 and drug 3TC, most of the FullFitness scores for protein variants with target drugs were larger than those of consensus RTs, indicating a decreased binding probability. These results were also validated in protein variants with three newly detected patterns (Figure 7). The docking results indicated that the mutation proteins that fit the detected drug-resistant patterns decreased the binding ability of the same target drugs.

## 4. Discussion

NRTIs are widely used to treat patients with HIV-1 infection by targeting the RT protein and functioning as a chain terminator in the viral DNA replication step. As a typical RNA virus, HIV mutated rapidly, which may cause drug resistance of previous NRTIs to mutated RT proteins. Thus, predicting the drug resistance between RT proteins and NRTIs could guide clinical medication and guide the broad-spectrum drug development of RT mutants. In this study, by incorporating the protein and molecule descriptors with a random forest classifier, we generated a PCM model to predict the drug resistance relationship between RT mutants and six FDA-approved NRTIs. Moreover, we detected the mutation patterns that may lead to drug resistance and validated the mutation patterns through previously reported drug-resistance experiments or molecular docking.

Currently, multiple models were constructed for drug resistance prediction using different protein and ligand descriptors [12,15]. Although most of them could achieve high prediction performance, the black box prediction obtained through the machine learning model makes it difficult to point out the mutation patterns that make great contributions to the model performance. Thus, besides prediction ability, we also tried to improve the interpretability of the model to clarify the changes in biological situations. To achieve that, two aspects have been considered in our model: (1) the important features contributing to the drug-resistance prediction were quantified, and both the important sites and important property changes were illustrated; (2) the mutation patterns were considered not only on the individual sites but also on the joint site fragments. The dominant joint peptides in drug-resistant protein could provide the sequence characters which may help the drug usage prediction in clinical.

In this study, we attempted to detect the mutation features that contribute to model performance, and all 47 mutation sites in drug-resistance mutants were detected. Among them, 10 dominant sites with top-ranked contributions were derived to generate the mutation pattern. The HIV Drug Resistance Database [18] proposed nine major drug resistance positions for NRTI, including sites 41, 65, 70, 74, 75, 151, 184, 210, and 215, five of which were detected in our ten dominant sites, indicating the importance of these positions for drug resistance. Moreover, 10 dominant sites were combined to form the mutation residue patterns of the resistance proteins. Validation through target-ligand docking indicated that proteins fitting the resistant mutation patterns resulted in decreased binding probability with NRTIs. Mutations in RT variants, including mutations that do not appear in the binding pocket, may involve changes in the physicochemical properties and spatial layout of the micro-environment, which will affect the binding between RT variants and NRTIs. The resistant mutation pattern detected in this model could be used in phenotype-testing prediction before clinical drug usage and screening of NRTIs.

## 5. Conclusions

This study introduced a PCM model to construct a drug resistance prediction model between mutated RT variants and available NRTIs. Through the random forest classifier, the relationship between drug resistance or susceptibility between RT variants and NRTIs could be predicted. Furthermore, the 10 dominant mutation sites on RT variants were detected, and the single or combined drug resistance patterns on the above dominant sites were revealed. This model could be applied to NRTI resistance evaluation in pre-clinical treatment and provide information for further RT-related therapy design.

## Figures and Tables

**Table 1:** The model performance of 10-fold cross-validation using different machine learning approaches on training dataset.

	AUC	Accuracy	F-Score	Precision	Recall
Random Forest	0.921 ± 0.060 *	0.827 ± 0.073	0.822 ± 0.087	0.815 ± 0.070	0.777 ± 0.206
Logistic Regression	0.871 ± 0.076	0.768 ± 0.094	0.758 ± 0.112	0.752 ± 0.104	0.750 ± 0.195
Decision Tree	0.791 ± 0.073	0.788 ± 0.069	0.793 ± 0.068	0.766 ± 0.070	0.772 ± 0.142
Naïve Bayes	0.813 ± 0.136	0.712 ± 0.099	0.685 ± 0.133	0.743 ± 0.121	0.596 ± 0.287
Supporting Vector Machine	0.896 ± 0.068	0.772 ± 0.098	0.758 ± 0.119	0.780 ± 0.107	0.717 ± 0.241

* Values refer to the mean and standard deviation of each result.

## Data Availability

Not applicable.

## Supplementary Materials

The following are available online at https://www.mdpi.com/article/10.3390/biom11091302/s1, Figure S1: Amino acid distribution and frequency changes of ABC-susceptible and ABC-resistant proteins on 47 sites, Figure S2: Amino acid distribution and frequency changes of AZT-susceptible and AZT-resistant proteins on 47 sites, Figure S3: Amino acid distribution and frequency changes of D4T-susceptible and D4T-resistant proteins on 47 sites, Figure S4: Amino acid distribution and frequency changes of DDI-susceptible and DDI-resistant proteins on 47 sites, Figure S5: Amino acid distribution and frequency changes of TDF-susceptible and TDF-resistant proteins on 47 sites, Table S1: The cutoff and range of fold resistance for each drugs, Table S2: Data distribution of different classes, Table S3: List of 47 mutation sites, Table S4: Z-scale for 20 amino acids, Table S5: Description of the 200-bit drug descriptors calculated by RDKit, Table S6: Hyper parameters of each tested machine learning models, Table S7: The detailed version of installed packages, Table S8: The nearest distance between 47 mutation sites and ligand binding atoms, Table S9: The *p*-value of Mann-Whitney test on the probability value for each class in 10-fold cross validation, Table S10: Model performance of the 10 times independent test, Table S11: List of top 40 ranked features, Table S12: *SD(s_j_)* values on 10 important sites and 37 mutation sites, Table S13: The *t*-test result between *SD(s_j_)* values on top 10 important sites and *SD(s_j_)* on 37 other mutation sites, Table S14: Binding ability between each drug and mutated protein variants for each detected mutation pattern.
