# Supplementary material for: Revealing the Mutation Patterns of Drug-Resistant Reverse Transcriptase Variants of Human Immunodeficiency Virus through Proteochemometric Modeling
Source: Biomolecules. 2021 Sep 2;11(9):1302. doi: 10.3390/biom11091302 (PMC8467226; doi:10.3390/biom11091302)
Supplement: Supplementary file 1 [file biomolecules-11-01302-s001.zip › biomolecules-1321450-supplementary.pdf]

biomolecules

MDPI

Supplementary

Table S1. The cutoff and range of fold resistance for each drugs.

drug	Cutoff for fold resistance[1]	The minimum of fold resistance	The maximum of fold resistance	Number of drug-susceptible pairs	Number of drug-resistant pairs
3TC	1.5	0.5	100	446	1192
ABC	4.5	0.5	100	978	595
AZT	2.2	0.1	100	846	813
D4T	1.7	0.4	100	1046	623
DDI	1.7	0.4	100	1071	598
TDF	1.5	0.2	100	930	400

Table S2. Data distribution of different classes.

Dataset	Susceptible variants	Resistant variants	Total
**Training dataset**	4250	3375	7625
**Testing dataset**	1067	846	1913

Table S3. List of 47 mutation sites.

Site	Site	Site	Site	Site	Site	Site	Site
20	35	39	41	43	44	60	67
69	70	74	83	98	101	102	103
118	122	123	135	142	162	174	177
178	179	181	184	190	196	200	202
203	207	208	210	211	214	215	219
228	245	272	277	286	293	297	

Table S4. Z-scale for 20 amino acids.

no.	abbrev	name	z1	z2	z3	z4	z5
1	Ala	alanine	0.24	-2.32	0.6	-0.14	1.3
2	Arg	arginine	3.52	2.5	-3.5	1.99	-0.17
3	Asn	asparagine	3.05	1.62	1.04	-1.15	1.61
4	Asp	aspartic acid	3.98	0.93	1.93	-2.46	0.75
5	Cys	cysteine	0.84	-1.67	3.71	0.18	-2.65
6	Gln	glutamine	1.75	0.5	-1.44	-1.34	0.66
7	Glu	glutamic acid	3.11	0.26	-0.11	-3.04	-0.25
8	Gly	glycine	2.05	-4.06	0.36	-0.82	-0.38
9	His	histidine	2.47	1.95	0.26	3.9	0.09
10	Ile	isoleucine	-3.89	-1.73	-1.71	-0.84	0.26
11	Leu	leucine	-4.28	-1.3	-1.49	-0.72	0.84
12	Lys	lysine	2.29	0.89	-2.49	1.49	0.31
13	Met	methionine	-2.85	-0.22	0.47	1.94	-0.98
14	Phe	phenylalanine	-4.22	1.94	1.06	0.54	-0.62
15	Pro	proline	-1.66	0.27	1.84	0.7	2
16	Ser	serine	2.39	-1.07	1.15	-1.39	0.67
17	Thr	threonine	0.75	-2.18	-1.12	-1.46	-0.4
18	Trp	tryptophan	-4.36	3.94	0.59	3.44	-1.59
19	Tyr	tyrosine	-2.54	2.44	0.43	0.04	-1.47
20	Val	valine	-2.59	-2.64	-1.54	-0.85	-0.02

Table S5. Description of the 200-bit drug descriptors calculated by RDKit.

NO	Name	Description
1	qed	Calculate the weighted sum of ADS mapped properties.
2	MinAbsPartialCharge	No specific description, calculated from the function of rdkit.Chem.Descriptors.MinAbsPartial-Charge(mol, force=False).
3	NumRadicalElectrons	The number of radical electrons the molecule has.
4	FpDensityMorgan1	No specific description, calculated from the function of rdkit.Chem.Descriptors.FpDensityMor-gan1(x).
5	FpDensityMorgan2	No specific description, calculated from the function of rdkit.Chem.Descriptors.FpDensityMor-gan2(x).
6	FpDensityMorgan3	No specific description, calculated from the function of rdkit.Chem.Descriptors.FpDensityMor-gan3(x).
7	MinPartialCharge	No specific description, calculated from the function of rdkit.Chem.Descriptors.MinPartial-Charge(mol, force=False).
8	MaxPartialCharge	No specific description, calculated from the function of rdkit.Chem.Descriptors.MaxPartial-Charge(mol, force=False).
9	HeavyAtomMolWt	The average molecular weight of the molecule ignoring hydrogens.
10~13	MaxAbsEStateIndex, MinEStateIndex, MinAbsEStateIndex, MaxEStateIndex	Basic EState definitions from the paper [Hall, Mohney and Kier. JCICS,31:76-81(1991)].
14	MaxAbsPartialCharge	No specific description, calculated from the function of rdkit.Chem.Descriptors.MaxAbsPartial-Charge(mol, force=False).
15	ExactMolWt	The exact molecular weight of the molecule.
16	MolWt	The average molecular weight of the molecule.
17	NumValenceElectrons	The number of valence electrons the molecule has.
18	BalabanJ	Calculate Balaban's J value for a molecule[Chem. Phys. Lett. 89:399-404 (1982)].
19	BertzCT	Complexity of the bonding, and the complexity of the distribution of heteroatoms.[J. Am. Chem. Soc. 103:3599-601 (1981)].
20~36	Chi0-Chi1, Chi0n-Chi4n, Chi0v-Chi4v, HallKierAlpha, Kappa1~Kappa3	Definitions from the paper[Rev. Comput. Chem. **2**:367-422 (1991)].
37	Ipc	The coefficients of the characteristic polynomial of the adjacency matrix of a hydrogen-sup-pressed graph of a molecule.
38	LabuteASA	Calculates Labute's Approximate Surface Area.
39~52	PEOE_VSA1~PEOE_VSA14	MOE-type descriptors using partial charges and surface area contributions http://www.chem-comp.com/journal/vsadesc.htm.
53~62	SMR_VSA1~SMR_VSA10	MOE-type descriptors using MR contributions and surface area contributions http://www.chem-comp.com/journal/vsadesc.htm.
63~73	SlogP_VSA1~SlogP_VSA12	MOE-type descriptors using LogP contributions and surface area contributions http://www.chemcomp.com/journal/vsadesc.htm.
74	TPSA	J. Med. Chem. **43**:3714-7, (2000).
75~95	EState_VSA1~EState_VSA11, VSA_EState1~VSA_EState10	MOE-type descriptors using EState indices and surface area contributions.
96	FractionCSP3	The fraction of carbons that are sp3 hybridized.
97	HeavyAtomCount	Number of heavy atoms a molecule.
98	NHOHCount	Number of NHs or Ohs.
99	NOCount	Number of Nitrogens and Oxygens.
100	NumAliphaticCarbocycles	The number of aliphatic (containing at least one non-aromatic bond) carbocycles for a molecule.
101	NumAliphaticHeterocycles	The number of aliphatic (containing at least one non-aromatic bond) heterocycles for a molecule.
102	NumAliphaticRings	The number of aliphatic (containing at least one non-aromatic bond) rings for a molecule.
103	NumAromaticCarbocycles	The number of aromatic carbocycles for a molecule.
104	NumAromaticHeterocycles	The number of aromatic heterocycles for a molecule.
105	NumAromaticRings	The number of aromatic rings for a molecule.
106	NumHAcceptors	Number of Hydrogen Bond Acceptors.
107	NumHDonors	Number of Hydrogen Bond Donors.
108	NumHeteroatoms	Number of Heteroatoms.
109	NumRotatableBonds	Number of Rotatable Bonds.
110	NumSaturatedCarbocycles	The number of saturated carbocycles for a molecule.
111	NumSaturatedHeterocycles	The number of saturated heterocycles for a molecule.
112	NumSaturatedRings	The number of saturated rings for a molecule.
113	RingCount	The query match the ring count.
114	MolLogP	Wildman and Crippen JCICS **39**:868-73 (1999).

115	MolMR	Wildman and Crippen JCICS **39**:868-73 (1999).
116	fr_Al_COO	Number of aliphatic carboxylic acids.
117	fr_Al_OH	Number of aliphatic hydroxyl groups.
118	fr_Al_OH_noTert	Number of aliphatic hydroxyl groups excluding tert-OH.
119	fr_ArN	Number of N functional groups attached to aromatics.
120	fr_Ar_COO	Number of Aromatic carboxylic acide.
121	fr_Ar_N	Number of aromatic nitrogens.
122	fr_Ar_NH	Number of aromatic amines.
123	fr_Ar_OH	Number of aromatic hydroxyl groups.
124	fr_COO	Number of carboxylic acids.
125	fr_COO2	Number of carboxylic acids.
126	fr_C_O	Number of carbonyl O.
127	fr_C_O_noCOO	Number of carbonyl O, excluding COOH.
128	fr_C_S	Number of thiocarbonyl.
129	fr_HOCCN	Number of C(OH)CCN-Ctert-alkyl or C(OH)CCNcyclic.
130	fr_Imine	Number of Imines.
131	fr_NH0	Number of Tertiary amines.
132	fr_NH1	Number of Secondary amines.
133	fr_NH2	Number of Primary amines.
134	fr_N_O	Number of hydroxylamine groups.
135	fr_Ndealkylation1	Number of XCCNR groups.
136	fr_Ndealkylation2	Number of tert-alicyclic amines (no heteroatoms, not quinine-like bridged N).
137	fr_Nhpyrrole	Number of H-pyrrole nitrogens.
138	fr_SH	Number of thiol groups.
139	fr_aldehyde	Number of aldehydes.
140	fr_alkyl_carbamate	Number of alkyl carbamates (subject to hydrolysis).
141	fr_alkyl_halide	Number of alkyl halides.
142	fr_allylic_oxid	Number of allylic oxidation sites excluding steroid dienone.
143	fr_amide	Number of amides.
144	fr_amidine	Number of amidine groups.
145	fr_aniline	Number of anilines.
146	fr_aryl_methyl	Number of aryl methyl sites for hydroxylation.
147	fr_azide	Number of azide groups.
148	fr_azo	Number of azo groups.
149	fr_barbitur	Number of barbiturate groups.
150	fr_benzene	Number of benzene rings.
151	fr_benzodiazepine	Number of benzodiazepines with no additional fused rings.
152	fr_bicyclic	Bicyclic.
153	fr_diazo	Number of diazo groups.
154	fr_dihydropyridine	Number of dihydropyridines.
155	fr_epoxide	Number of epoxide rings.
156	fr_ester	Number of esters.
157	fr_ether	Number of ether oxygens (including phenoxy).
158	fr_furan	Number of furan rings.
159	fr_guanido	Number of guanidine groups.
160	fr_halogen	Number of halogens.
161	fr_hdrzine	Number of hydrazine groups.
162	fr_hdrzone	Number of hydrazone groups.
163	fr_imidazole	Number of imidazole rings.
164	fr_imide	Number of imide groups.
165	fr_isocyan	Number of isocyanates.
166	fr_isothiocyan	Number of isothiocyanates.
167	fr_ketone	Number of ketones.
168	fr_ketone_Topliss	Number of ketones excluding diaryl, a,b-unsat. dienones, heteroatom on Calpha.
169	fr_lactam	Number of beta lactams.
170	fr_lactone	Number of cyclic esters (lactones).
171	fr_methoxy	Number of methoxy groups -OCH3.
172	fr_morpholine	Number of morpholine rings.
173	fr_nitrile	Number of nitriles.
174	fr_nitro	Number of nitro groups.
175	fr_nitro_arom	Number of nitro benzene ring substituents.
176	fr_nitro_arom_nonortho	Number of non-ortho nitro benzene ring substituents.
177	fr_nitroso	Number of nitroso groups, excluding NO2.
178	fr_oxazole	Number of oxazole rings.
179	fr_oxime	Number of oxime groups.
180	fr_para_hydroxylation	Number of para-hydroxylation sites.

181	fr_phenol	Number of phenols.
182	fr_phenol_noOrthoHbond	Number of phenolic OH excluding ortho intramolecular Hbond substituents.
183	fr_phos_acid	Number of phosphoric acid groups.
184	fr_phos_ester	Number of phosphoric ester groups.
185	fr_piperdine	Number of piperdine rings.
186	fr_piperzine	Number of piperzine rings.
187	fr_priamide	Number of primary amides.
188	fr_prisulfonamd	Number of primary sulfonamides.
189	fr_pyridine	Number of pyridine rings.
190	fr_quatN	Number of quarternary nitrogens.
191	fr_sulfide	Number of thioether.
192	fr_sulfonamd	Number of sulfonamides.
193	fr_sulfone	Number of sulfone groups.
194	fr_term_acetylene	Number of terminal acetylenes.
195	fr_tetrazole	Number of tetrazole rings.
196	fr_thiazole	Number of thiazole rings.
197	fr_thiocyan	Number of thiocyanates.
198	fr_thiophene	Number of thiophene rings.
199	fr_unbrch_alkane	Number of unbranched alkanes of at least 4 members (excludes halogenated alkanes).
200	fr_urea	Number of urea groups.

Table S6. Hyper parameters of each tested machine learning models.

**Machine learning approaches**	**hyper parameters**
Random Forest*	'warm_start': False,<br>'oob_score': False,<br>'n_jobs': None,<br>'min_impurity_decrease': 0.0,<br>'verbose': 0,<br>'max_leaf_nodes': None,<br>'bootstrap': True,<br>'min_samples_leaf': 1,<br>'n_estimators': 67,<br>'min_samples_split': 4,<br>'min_weight_fraction_leaf': 0.0,<br>'criterion': 'gini',<br>'random_state': None,<br>'min_impurity_split': None,<br>'max_features': 27,<br>'max_depth': 20,<br>'class_weight': None
Logistic Regression	'warm_start': False,<br>'C': 1.0,<br>'n_jobs': None,<br>'verbose': 0,<br>'intercept_scaling': 1,<br>'fit_intercept': True,<br>'max_iter': 100,<br>'penalty': 'l2',<br>'multi_class': 'warn',<br>'random_state': None,<br>'dual': False,<br>'tol': 0.0001,<br>'solver': 'warn',<br>'class_weight': None
Decision Tree	'presort': False,<br>'splitter': 'best',<br>'min_impurity_decrease': 0.0,<br>'max_leaf_nodes': None,<br>'min_samples_leaf': 1,<br>'min_samples_split': 2,<br>'min_weight_fraction_leaf': 0.0,

	'criterion': 'gini',<br>'random_state': None,<br>'min_impurity_split': None,<br>'max_features': None,<br>'max_depth': None,<br>'class_weight': None
Naive Bayes	'priors': None,<br>'var_smoothing': 1e-09
Support Vector Machine	'kernel': 'rbf',<br>'C': 1.0,<br>'verbose': False,<br>'probability': False,<br>'degree': 3,<br>'shrinking': True,<br>'max_iter': -1,<br>'decision_function_shape': 'ovr',<br>'random_state': None,<br>'tol': 0.001,<br>'cache_size': 200,<br>'coef0': 0.0,<br>'gamma': 'auto_deprecated',<br>'class_weight': None

*The hyper parameters for Random Forest was optimized by GridSearchCV function in scikit-learn module and parameters for other approaches were the default in scikit-learn module.

Table S7. The detailed version of installed packages.

Package	Version
certifi	2020.4.5.1
joblib	0.14.1
mkl-fft	1.0.15
mkl-random	1.1.0
mkl-service	2.3.0
numpy	1.18.1
pip	20.0.2
scikit-learn	0.22.1
scipy	1.4.1
setuptools	46.2.0.post20200511
six	1.14.0
wheel	0.34.2
wincertstore	0.2

Table S8. The nearest distance between 47 mutation sites and ligand binding atoms.

NO.	47 mutation site in consensus RT	Nearest ligand binding atom	Ligand binding region	Distance(Å)
1	181	124	A	2.102
2	184	597	B	2.667
3	272	816	D	2.963
4	74	723	C	3.381
5	162	348	A	4.377
6	98	129	A	5.587
7	67	691	C	5.616
8	101	124	A	6.675
9	70	726	C	6.980
10	179	124	A	7.164
11	293	686	C	7.775

12	245	657	C	7.923
13	60	751	C	8.446
14	118	548	B	8.809
15	286	686	C	9.377
16	69	726	C	9.718
17	83	455	B	9.767
18	214	548	B	9.774
19	102	124	A	10.254
20	103	124	A	10.414
21	178	312	A	10.925
22	190	350	A	11.586
23	41	653	B	12.247
24	177	124	A	12.488
25	228	296	A	12.491
26	208	334	A	13.419
27	215	597	B	13.851
28	277	119	A	14.420
29	202	350	A	14.992
30	174	334	A	15.274
31	44	646	B	15.827
32	35	751	C	16.247
33	123	455	B	17.032
34	122	489	B	17.039
35	210	456	B	17.077
36	219	634	B	17.924
37	20	725	C	18.058
38	207	350	A	18.26
39	135	751	C	18.549
40	203	350	A	18.567
41	39	732	C	18.635
42	211	334	A	18.706
43	142	751	C	18.813
44	297	657	C	19.023
45	200	350	A	20.375
46	43	646	B	20.553
47	196	350	A	23.806

Table S9. The p-value of Mann-Whitney test on the probability value for each class in 10-fold cross validation.

Machine learning approaches	Cross validation	Number of susceptible pairs	Number of resistant pairs	p-value
Random Forest	1	413	349	1.62E-80
	2	426	336	5.24E-82
	3	426	336	1.56E-88
	4	427	335	4.47E-82
	5	422	340	1.42E-91
	6	435	327	1.41E-85
	7	418	344	4.46E-81
	8	444	318	1.21E-86
	9	430	332	8.39E-84
	10	407	355	1.80E-84
	total	4250	3375	0.00
Logistic Regression	1	413	349	3.22E-53
	2	426	336	1.92E-58
	3	426	336	6.32E-57
	4	427	335	7.10E-59
	5	422	340	3.03E-65
	6	435	327	4.39E-58
	7	418	344	5.36E-53
	8	444	318	1.10E-61
	9	430	332	4.25E-63
	10	407	355	3.41E-55
	total	4250	3375	0.00

Decision Tree	1	413	349	1.88E-51
	2	426	336	4.92E-54
	3	426	336	2.14E-58
	4	427	335	2.87E-55
	5	422	340	2.33E-60
	6	435	327	1.67E-59
	7	418	344	2.13E-53
	8	444	318	4.69E-55
	9	430	332	2.60E-59
	10	407	355	1.96E-57
	total	4250	3375	0.00
Naïve Bayes	1	413	349	7.56E-23
	2	426	336	6.35E-19
	3	426	336	2.07E-23
	4	427	335	3.01E-24
	5	422	340	7.43E-29
	6	435	327	3.04E-29
	7	418	344	1.79E-20
	8	444	318	7.95E-23
	9	430	332	2.13E-29
	10	407	355	6.66E-25
	total	4250	3375	4.90E-229
Supporting Vector Machine*	1	413	349	4.35E-44
	2	426	336	2.08E-47
	3	426	336	1.11E-49
	4	427	335	2.68E-49
	5	422	340	2.38E-53
	6	435	327	4.01E-50
	7	418	344	1.79E-41
	8	444	318	4.09E-51
	9	430	332	2.22E-58
	10	407	355	9.12E-50
	total	4250	3375	0.00

*Supporting vector machine approach could not provide the prediction probability during classification, alternatively the predicted binary class result was used in this part of test.

Table S10. Model performance of the 10 times independent test.

	**AUC**	**Accuracy**	**F-score**	**Precision**	**Recall**
**Independent_split_1**	0.945	0.866	0.866	0.867	0.866
**Independent_split_2**	0.941	0.866	0.865	0.865	0.866
**Independent_split_3**	0.946	0.869	0.869	0.869	0.869
**Independent_split_4**	0.948	0.873	0.873	0.873	0.873
**Independent_split_5**	0.939	0.866	0.866	0.866	0.866
**Independent_split_6**	0.951	0.880	0.880	0.880	0.880
**Independent_split_7**	0.946	0.874	0.874	0.874	0.874
**Independent_split_8**	0.952	0.876	0.876	0.876	0.876
**Independent_split_9**	0.944	0.875	0.875	0.876	0.875
**Independent_split_10**	0.948	0.886	0.885	0.885	0.886
**Mean**	0.946	0.873	0.873	0.873	0.873
**Standard Deviation**	0.004	0.007	0.007	0.006	0.007

Table S11. List of top 40 ranked features.

**Rank**	**Feature**	**Sorted importance**	**Accumulated importance**	**Growth rate**
1	210_z2	0.031	0.031	-
2	215_z2	0.027	0.058	0.881
3	215_z3	0.024	0.082	0.416
4	41_z2	0.024	0.106	0.287
5	67_z2	0.021	0.127	0.201

6	215_z1	0.021	0.149	0.166
7	215_z5	0.020	0.169	0.134
8	215_z4	0.019	0.187	0.111
9	210_z5	0.018	0.205	0.095
10	41_z4	0.018	0.222	0.086
11	184_z5	0.017	0.239	0.076
12	41_z5	0.016	0.255	0.065
13	184_z2	0.015	0.270	0.059
14	67_z5	0.015	0.285	0.055
15	210_z1	0.015	0.300	0.052
16	184_z3	0.015	0.315	0.049
17	184_z1	0.014	0.329	0.046
18	41_z3	0.014	0.343	0.043
19	184_z4	0.013	0.356	0.039
20	41_z1	0.013	0.369	0.036
21	210_z4	0.011	0.380	0.031
22	67_z3	0.008	0.389	0.022
23	67_z4	0.008	0.397	0.020
24	70_z1	0.008	0.404	0.019
25	118_z4	0.007	0.411	0.018
26	219_z5	0.007	0.418	0.017
27	219_z3	0.007	0.425	0.017
28	FpDensityMorgan2	0.007	0.432	0.015
29	219_z2	0.006	0.438	0.015
30	70_z5	0.006	0.444	0.014
31	SMR_VSA5	0.006	0.451	0.014
32	70_z4	0.006	0.457	0.013
33	219_z1	0.006	0.463	0.013
34	70_z2	0.006	0.468	0.013
35	SlogP_VSA1	0.006	0.474	0.012
36	228_z1	0.005	0.479	0.012
37	67_z1	0.005	0.485	0.011
38	VSA_EState9	0.005	0.490	0.011
39	FpDensityMorgan1	0.005	0.495	0.010
40	69_z4	0.005	0.500	0.010

Table S12. *SD(s_j)* values on 10 important sites and 37 mutation sites.

	**sites**	**Drug**					
		**3TC**	**ABC**	**AZT**	D4T	DDI	TDF
10 dominant sites	41	0.140	0.165	0.194	0.184	0.111	0.145
	67	0.125	0.107	0.156	0.149	0.087	0.116
	69	0.047	0.038	0.055	0.064	0.050	0.053
	70	0.063	0.026	0.080	0.048	0.016	0.054
	118	0.067	0.096	0.101	0.121	0.094	0.080
	184	0.191	0.156	0.062	0.035	0.103	0.009
	210	0.101	0.137	0.144	0.160	0.103	0.120
	215	0.153	0.159	0.201	0.173	0.105	0.129
	219	0.085	0.070	0.096	0.083	0.055	0.074
	228	0.051	0.052	0.066	0.074	0.053	0.064
37 mutation sites	20	0.015	0.022	0.023	0.025	0.022	0.008
	35	0.025	0.010	0.016	0.009	0.010	0.013
	39	0.035	0.043	0.045	0.056	0.041	0.041
	43	0.045	0.060	0.065	0.077	0.057	0.064
	44	0.040	0.058	0.060	0.073	0.056	0.049
	60	0.001	0.017	0.023	0.027	0.010	0.040
	74	0.043	0.057	0.034	0.044	0.066	0.012
	83	0.039	0.031	0.021	0.022	0.028	0.017
	98	0.021	0.024	0.025	0.025	0.014	0.040
	101	0.017	0.016	0.017	0.020	0.022	0.019
	102	0.005	0.006	0.010	0.006	0.003	0.015
	103	0.037	0.023	0.030	0.030	0.031	0.028
	122	0.040	0.057	0.050	0.055	0.040	0.038
	123	0.006	0.006	0.010	0.007	0.005	0.011

135	0.014	0.033	0.033	0.032	0.022	0.042
142	0.016	0.012	0.014	0.011	0.014	0.004
162	0.011	0.017	0.017	0.017	0.014	0.012
174	0.011	0.006	0.009	0.005	0.004	0.003
177	0.013	0.003	0.005	0.005	0.008	0.008
178	0.014	0.012	0.014	0.011	0.012	0.007
179	0.018	0.020	0.020	0.023	0.017	0.014
181	0.044	0.019	0.020	0.029	0.036	0.009
190	0.029	0.024	0.026	0.032	0.025	0.030
196	0.017	0.026	0.019	0.026	0.022	0.019
200	0.008	0.008	0.008	0.011	0.009	0.009
202	0.005	0.001	0.001	0.002	0.003	0.005
203	0.033	0.039	0.046	0.053	0.034	0.039
207	0.012	0.010	0.013	0.017	0.013	0.018
208	0.040	0.055	0.055	0.070	0.055	0.047
211	0.021	0.028	0.028	0.038	0.016	0.024
214	0.005	0.012	0.017	0.018	0.002	0.010
245	0.017	0.013	0.012	0.008	0.005	0.011
272	0.018	0.002	0.010	0.003	0.005	0.006
277	0.011	0.018	0.022	0.021	0.010	0.026
286	0.007	0.003	0.007	0.010	0.004	0.011
293	0.024	0.015	0.018	0.017	0.003	0.014
297	0.014	0.014	0.020	0.013	0.008	0.014

Table S13. The t-test result between *SD*(s_j) values on top 10 important sites and *SD*(s_j) on 37 other mutation sites.

Drug	p-value	Drug	p-value
3TC	4.59×10^{-4}**	D4T	9.21×10^{-4}**
ABC	1.01×10^{-3}**	DDI	2.33×10^{-4}**
AZT	4.30×10^{-4}**	TDF	9.96×10^{-4}**

**statistically significant (p value < 0.01).

Table S14. Binding ability between each drug and mutated protein variants for each detected mutation pattern.

Drug	Protein	mutation pattern#	Top1 FullFiteness	Relative FullFitness*	New pattern detected in predicted drug-resistant proteins&
3TC	variant 1	LDTKVVLYKL	-3655.82	11.051	
3TC	variant 2	LDTKVVWYKL	-3658.164	8.707	
3TC	variant 3	MDTKVVLTKL	-3666.981	-0.11	
3TC	consensus RT	MDTKVMLTKL	-3666.871	0	
ABC	variant 1	LNTKIVWYNL	-3479.942	50.632	New pattern
ABC	consensus RT	MDTKVMLTKL	-3530.574	0	
AZT	variant 1	LDTKVVLYKL	-3676.221	8.661	
AZT	consensus RT	MDTKVMLTKL	-3684.882	0	
TDF	variant 1	LDTKVVWYKL	-3692.176	6.845	
TDF	variant 2	LNTKIMWYKL	-3684.688	14.333	New pattern
TDF	variant 3	LNDKIVWYKL	-3693.079	5.942	New pattern
TDF	variant 4	LNTKIMWYRL	-3676.074	22.947	
TDF	variant 5	LNTKVVWYKL	-3697.397	1.624	
TDF	variant 6	LNTKIVWYKL	-3683.263	15.758	
TDF	consensus RT	MDTKVMLTKL	-3699.021	0	

#Mutation patterns were the joint fragment of residues on sites 41,67,69,70,118,184,210,215,219,228 on RT sequence. *The Relative Fullfitness score was calculated as the difference between the raw Fullfitness score of the drug-mutated protein and the raw Fullfitness score of the corresponding drug-consenses RT. &The new pattern detected in predicted drug-resistant proteins listed the protein patterns that have not been occurred in the drug-protein pairs with experiment result.

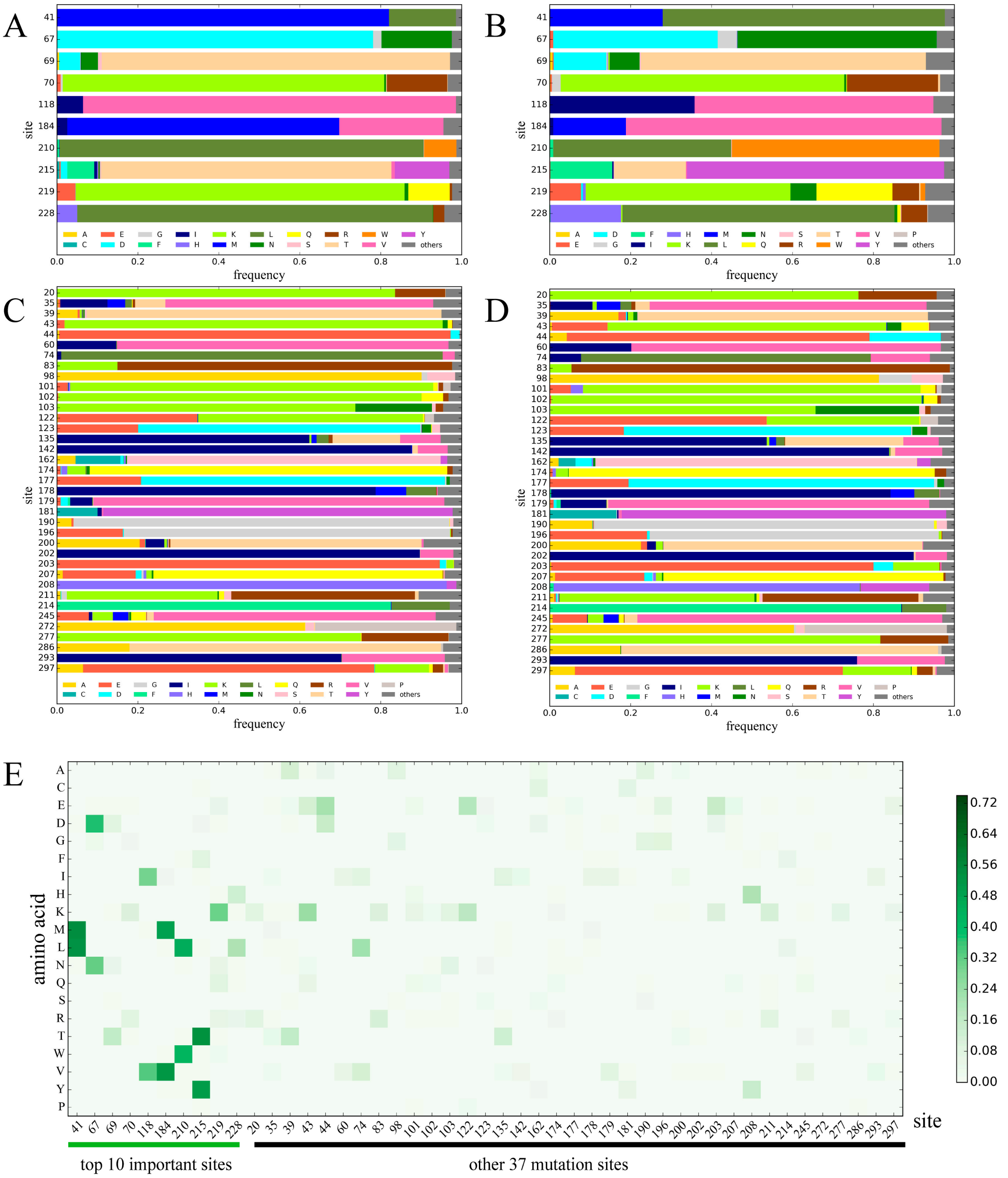

Figure S1. Amino acid distribution and frequency changes of ABC-susceptible and ABC-resistant proteins on 47 sites. Sub-graphs A and B show the amino acid frequency on 10 dominant sites for ABC-susceptible proteins and ABC-resistant proteins respectively. Sub-graphs C and D show the amino acid frequency on 37 mutation sites for ABC-susceptible proteins and ABC-resistant proteins respectively. Sub-graph E illustrates the absolute difference of residue frequency on each sites between ABC-susceptible and ABC-resistant proteins. X axis refers to each mutation sites. Y axis refers to 20 amino acids. Each pixel refers to the value of $DF(a_i,s_j)$ for each amino acid a_i on 47 sites s_j. The color of each pixel correlate to the value of $DF(a_i,s_j)$.

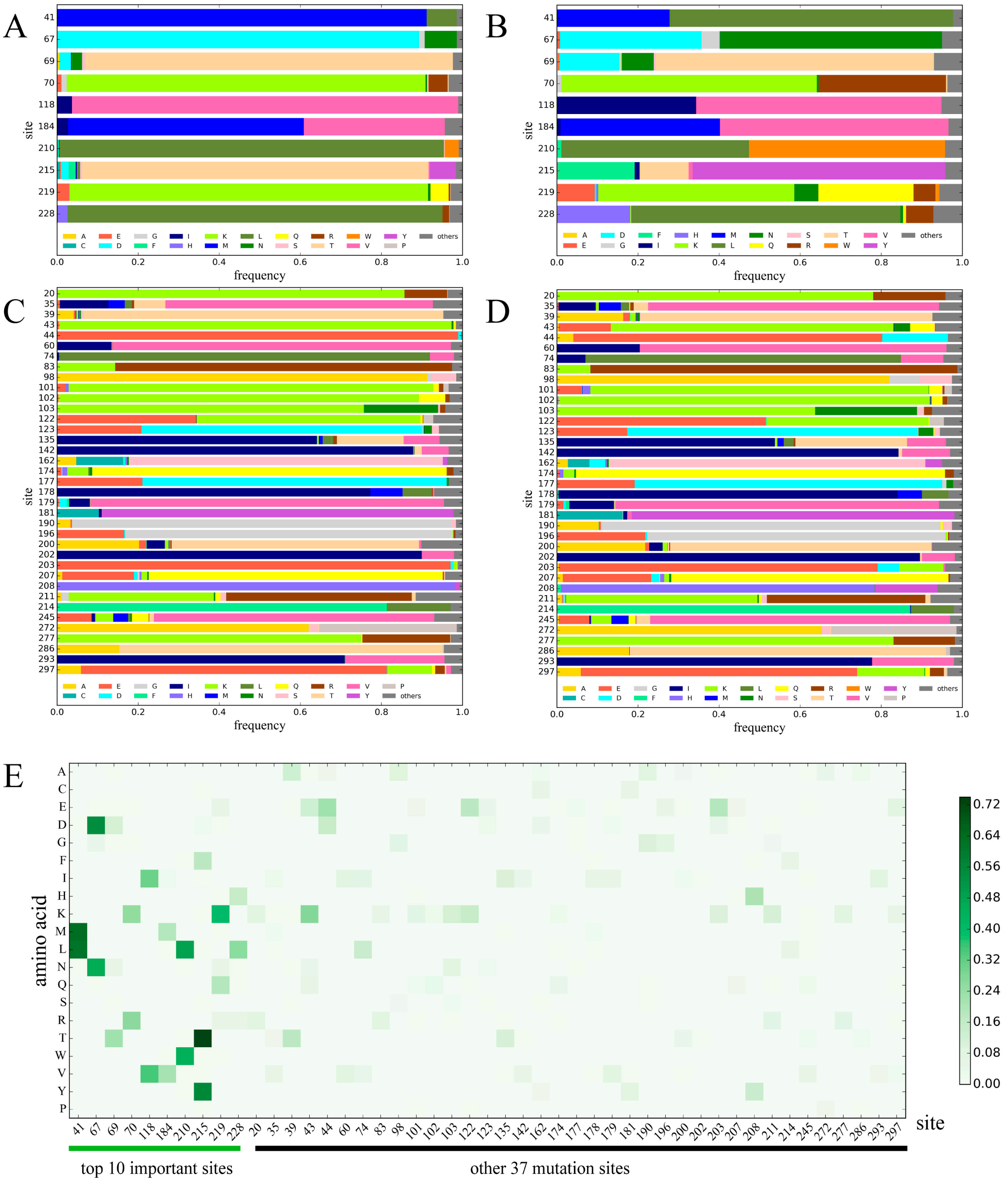

Figure S2. Amino acid distribution and frequency changes of AZT-susceptible and AZT-resistant proteins on 47 sites. Sub-graphs A and B show the amino acid frequency on 10 dominant sites for AZT-susceptible proteins and AZT-resistant proteins respectively. Sub-graphs C and D show the amino acid frequency on 37 mutation sites for AZT-susceptible proteins and AZT-resistant proteins respectively. Sub-graph E illustrates the absolute difference of residue frequency on each sites between AZT-susceptible and AZT-resistant proteins. X axis refers to each mutation sites. Y axis refers to 20 amino acids. Each pixel refers to the value of $DF(a_i,s_j)$ for each amino acid a_i on 47 sites s_j. The color of each pixel correlate to the value of $DF(a_i,s_j)$.

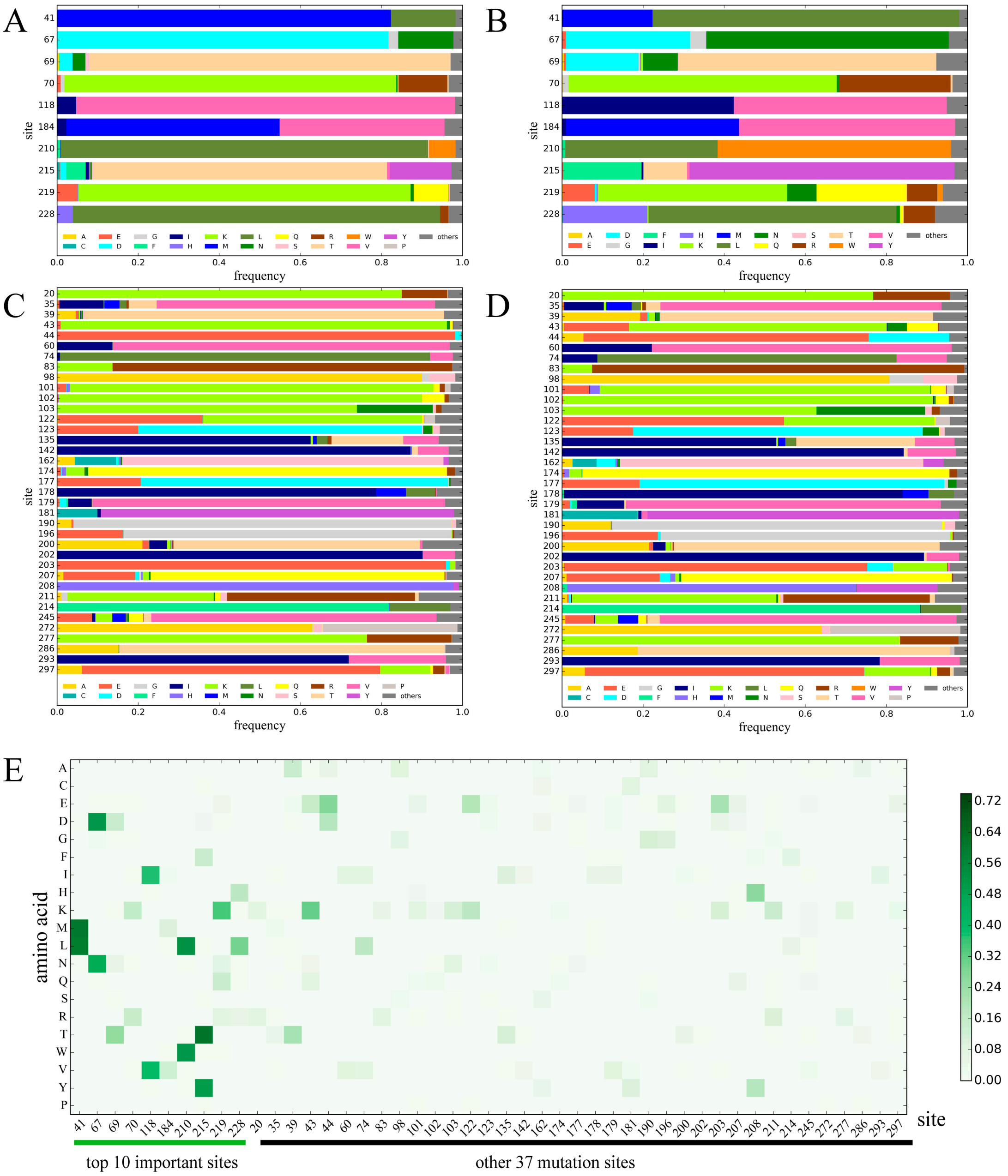

Figure S3. Amino acid distribution and frequency changes of D4T-susceptible and D4T-resistant proteins on 47 sites. Subgraphs A and B show the amino acid frequency on 10 dominant sites for D4T-susceptible proteins and D4T-resistant proteins respectively. Sub-graphs C and D show the amino acid frequency on 37 mutation sites for D4T-susceptible proteins and D4T-resistant proteins respectively. Sub-graph E illustrates the absolute difference of residue frequency on each sites between D4T-susceptible and D4T-resistant proteins. X axis refers to each mutation sites. Y axis refers to 20 amino acids. Each pixel refers to the value of $DF(a_i,s_j)$ for each amino acid a_i on 47 sites s_j. The color of each pixel correlate to the value of $DF(a_i,s_j)$.

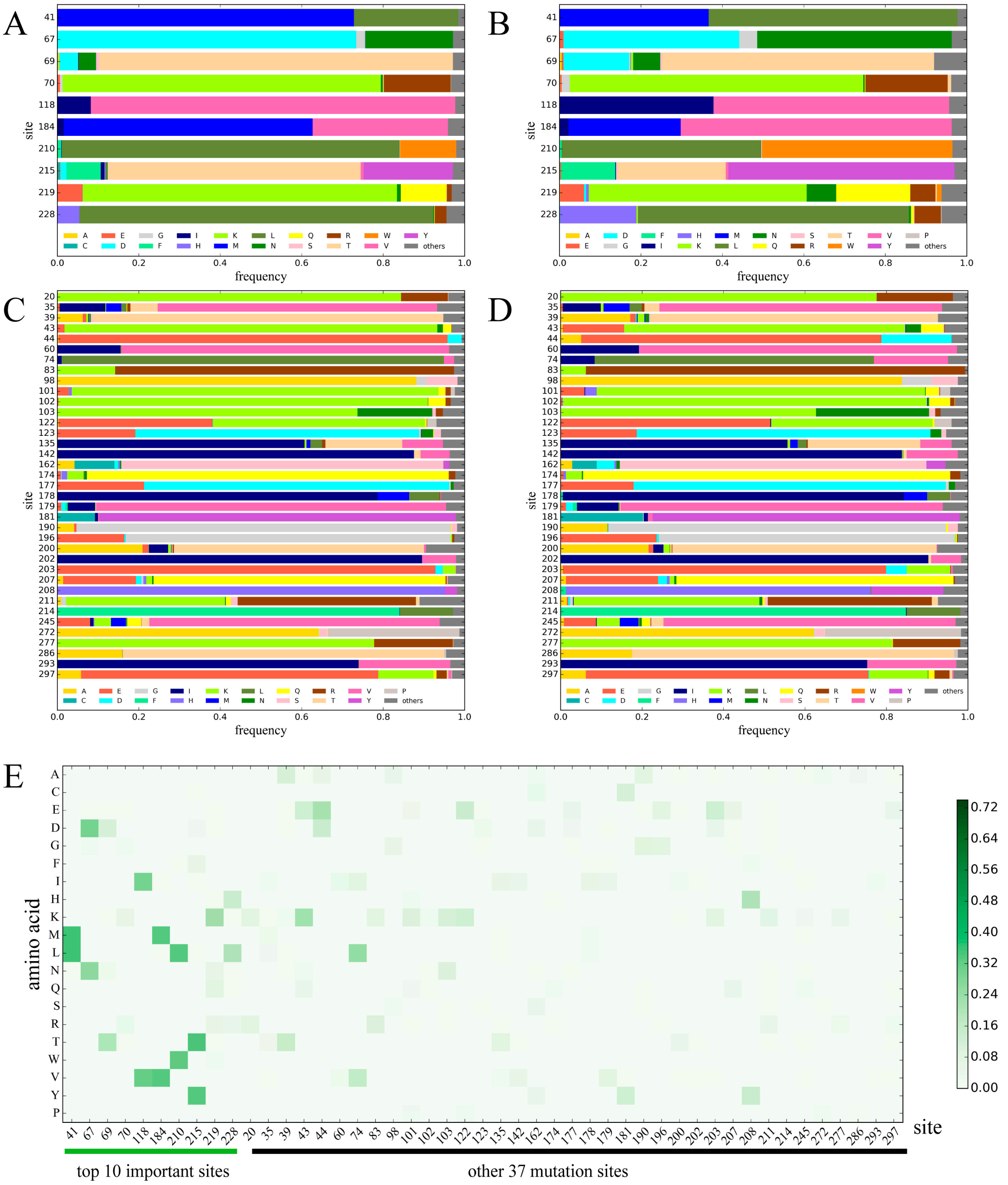

Figure S4. Amino acid distribution and frequency changes of DDI-susceptible and DDI-resistant proteins on 47 sites. Sub-graphs A and B show the amino acid frequency on 10 dominant sites for DDI-susceptible proteins and DDI-resistant proteins respectively. Sub-graphs C and D show the amino acid frequency on 37 mutation sites for DDI-susceptible proteins and DDI-resistant proteins respectively. Sub-graph E illustrates the absolute difference of residue frequency on each sites between DDI-susceptible and DDI-resistant proteins. X axis refers to each mutation sites. Y axis refers to 20 amino acids. Each pixel refers to the value of $DF(a_i,s_j)$ for each amino acid a_i on 47 sites s_j. The color of each pixel correlate to the value of $DF(a_i,s_j)$.

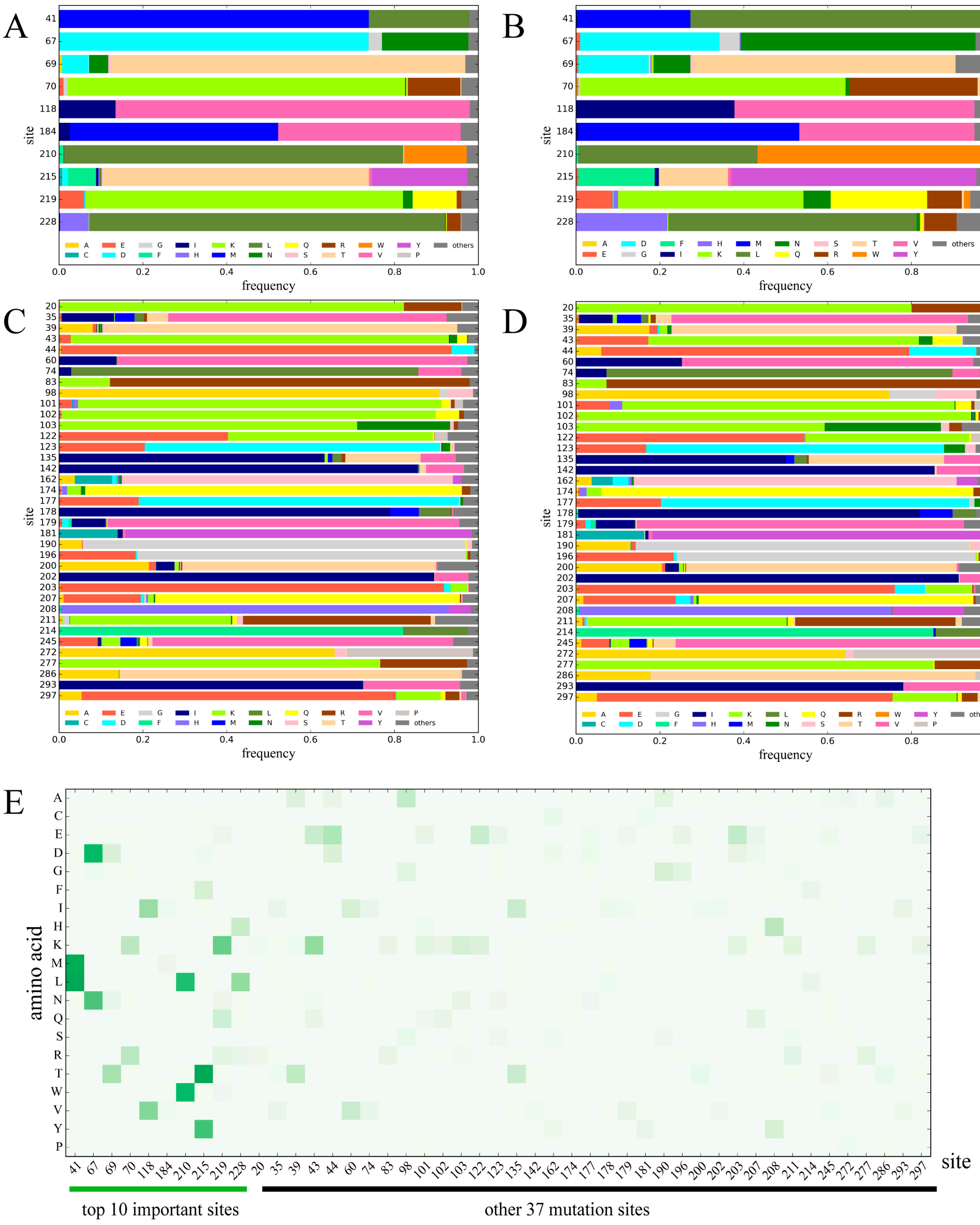

Figure S5. Amino acid distribution and frequency changes of TDF-susceptible and TDF-resistant proteins on 47 sites. Subgraphs A and B show the amino acid frequency on 10 dominant sites for TDF-susceptible proteins and TDF-resistant proteins respectively. Sub-graphs C and D show the amino acid frequency on 37 mutation sites for TDF-susceptible proteins and TDF-resistant proteins respectively. Sub-graph E illustrates the absolute difference of residue frequency on each sites between TDF-susceptible and TDF-resistant proteins. X axis refers to each mutation sites. Y axis refers to 20 amino acids. Each pixel refers to the value of $DF(a_i,s_j)$ for each amino acid a_i on 47 sites s_j. The color of each pixel correlate to the value of $DF(a_i,s_j)$.

Reference:

1. O. Tarasova, N. Biziukova, D. Filimonov, and V. Poroikov, "A Computational Approach for the Prediction of HIV Resistance Based on Amino Acid and Nucleotide Descriptors," Molecules, vol. 23, no. 11, Nov, 2018.
